# Street characteristics preferred for transportation walking among older adults: a choice-based conjoint analysis with manipulated photographs

**DOI:** 10.1186/s12966-016-0331-8

**Published:** 2016-01-16

**Authors:** Jelle Van Cauwenberg, Ilse De Bourdeaudhuij, Peter Clarys, Jack Nasar, Jo Salmon, Liesbet Goubert, Benedicte Deforche

**Affiliations:** Department of Public Health, Faculty of Medicine and Health Sciences, Ghent University, De Pintelaan 185, B-9000 Ghent, Belgium; Department of Human Biometry and Biomechanics, Faculty of Physical Education and Physical Therapy, Vrije Universiteit Brussel, Pleinlaan 2, B-1050 Brussels, Belgium; Research Foundation - Flanders (FWO), Egmontstraat 5, B-1000 Brussels, Belgium; Department of Movement and Sport Sciences, Faculty of Medicine and Health Sciences, Ghent University, Watersportlaan 2, B-9000 Ghent, Belgium; City and Regional Planning, The Ohio State University, 230 Knowlton Hall, Columbus, OH 43210 USA; School of Exercise & Nutrition Sciences, Deakin University, Burwood Highway 221, Burwood, Victoria 3125 Australia; Department of Experimental – Clinical and Health Psychology, Faculty of Psychology and Educational Sciences, Ghent University, Henry Dunantlaan 2, B-9000 Ghent, Belgium

**Keywords:** physical activity, mobility, active ageing, environment, ecological model, experiment, latent class analysis, service flat residents, seniors

## Abstract

**Background:**

Knowledge about the relationships between micro-scale environmental factors and older adults’ walking for transport is limited and inconsistent. This is probably due to methodological limitations, such as absence of an accurate neighborhood definition, lack of environmental heterogeneity, environmental co-variation, and recall bias. Furthermore, most previous studies are observational in nature. We aimed to address these limitations by investigating the effects of manipulating photographs on micro-scale environmental factors on the appeal of a street for older adults’ transportation walking. Secondly, we used latent class analysis to examine whether subgroups could be identified that have different environmental preferences for transportation walking. Thirdly, we investigated whether these subgroups differed in socio-demographic, functional and psychosocial characteristics, current level of walking and environmental perceptions of their own street.

**Methods:**

Data were collected among 1131 Flemish older adults through an online (*n* = 940) or an interview version of the questionnaire (*n* = 191). This questionnaire included a choice-based conjoint exercise with manipulated photographs of a street. These manipulated photographs originated from one panoramic photograph of an existing street that was manipulated on nine environmental attributes. Participants chose which of two presented streets they would prefer to walk for transport.

**Results:**

In the total sample, sidewalk evenness had by far the greatest appeal for transportation walking. The other environmental attributes were less important. Four subgroups that differed in their environmental preferences for transportation walking were identified. In the two largest subgroups (representing 86 % of the sample) sidewalk evenness was the most important environmental attribute. In the two smaller subgroups (each comprising 7 % of the sample), traffic volume and speed limit were the most important environmental attributes for one, and the presence of vegetation and a bench were the most important environmental attributes for the other. This latter subgroup included a higher percentage of service flat residents than the other subgroups.

**Conclusions:**

Our results suggest that the provision of even sidewalks should be considered a priority when developing environmental interventions aiming to stimulate older adults’ transportation walking. Natural experiments are needed to confirm whether our findings can be translated to real environments and actual transportation walking behavior.

**Electronic supplementary material:**

The online version of this article (doi:10.1186/s12966-016-0331-8) contains supplementary material, which is available to authorized users.

## Background

The physically inactive lifestyle of the majority of Western older adults (≥65 years) puts them at increased risk for morbidity and mortality [1–4]. Therefore, the promotion of physical activity (PA) among older adults is deemed crucial to foster healthy ageing [5]. The promotion of walking seems particularly promising since walking is a healthy [6, 7], accessible and well-liked [8] type of physical activity among older adults. Furthermore, walking for transport (e.g. to a shop or to a friend’s house) should be easy to integrate into the daily routines of most older adults.

According to socio-ecological models, the choice to walk for transport is not only determined by individual characteristics (such as attitudes and self-efficacy) but also by the environment in which older adults live [9, 10]. Having good access to a variety of daily destinations (such as grocery stores, bank offices, parks and libraries) has been linked consistently to higher levels of walking for transport among older adults [11–15]. Although easy access to daily destinations is important to stimulate older adults’ walking for transportation, these are macro-scale environmental factors that typically require high level government policy to change and come at a substantial economic cost. Hence, access to daily destinations is difficult to change in existing neighborhoods [16].

Micro-scale environmental factors (e.g. sidewalk characteristics and presence of vegetation), are mostly under the jurisdiction of local government and are more amenable to change [16]. Several qualitative studies suggested that micro-scale environmental factors are important for older adults’ walking for transport [17–20]. However, previous quantitative studies examining the cross-sectional relationships between micro-scale environmental factors and older adults’ transportation walking have yielded inconsistent findings [21, 22]. These inconsistencies might have been caused by the following methodological issues: no consensus about how to define a ‘local neighborhood’ for older adults [23, 24], recall issues when older adults respond to questionnaires targeting their environmental perceptions and experiences while not being in that environment [25], and limited variation in the environments being studied [26]. Furthermore, there is a tendency of environmental factors to co-occur (so-called environmental co-variation), which hinders differentiating the separate influence of each environmental factor [27]. Lastly, the vast majority of evidence in this research area comes from observational studies and there is a strong need for experimental research to establish causal associations [21, 28, 29]. However, conducting natural experiments in real environments is very expensive and time-consuming and such experiments may have long-lasting effects. Therefore, they should be well-informed to avoid unanticipated (negative) effects on older adults’ transportation walking.

This information may be obtained from studies using photographed street environments to examine the effects of manipulating micro-scale environmental factors on a streets’ appeal for walking for transport. This approach allows the researcher to observe the effects of hypothetical environmental changes on preferences for transportation walking under controlled conditions since it is easy to control the variation within and co-variation between the manipulated environmental factors. Furthermore, there is no need to accurately define an older adults’ ‘local neighborhood’ nor do the older adults have to recall their environmental perceptions and experiences because exposure to and assessment of the environment occurs simultaneously and consistently between participants. Results of such experiments with photographs can inform natural experiments about which environmental modifications will most likely stimulate older adults’ walking for transport leading to more effective natural experiments.

The use of manipulated photographed streets has been previously pilot-tested among 60 Belgian older adults [30]. The older adults sorted two sets of 16 photographs according to the streets’ appeal for transportation walking. Within each of the two sets of photographs four environmental attributes (six different environmental attributes in total) with two levels within each attribute (e.g. even versus uneven sidewalk) were manipulated. Sidewalk evenness was the most important environmental feature, which was even stronger when the street’s overall upkeep was good, there was presence of vegetation, and when traffic was absent. A limitation of that study (apart from the small sample size) was that only four environmental attributes with two levels were examined simultaneously. The inclusion of more than two levels of manipulation within an attribute will enable testing of whether a street’s appeal increases linearly with improvements in an environmental attribute or whether there exists a threshold after which further improvements in the environmental factor no longer result in increases in appeal. Therefore, in the current study we wanted to examine a larger set of environmental attributes with more levels of manipulation (e.g. even, slightly uneven and very uneven sidewalk). For this purpose, we used conjoint analysis which allows for numerous attributes and levels to be studied by assigning their combinations randomly across participants [31].

The importance of certain micro-scale environmental factors for walking for transport might differ between subgroups of the older population (based on socio-demographic and functional characteristics). In our previous pilot study it was found that compared to older adults who walk less than an hour per week, those who walk an hour or more reported that streets with benches were more inviting for walking [30]. Knowledge of such moderators is necessary for designing environments that suit the needs of multiple subgroups [32]. For example, press-competence models assume that when people become more functionally limited and their competence decreases, their sensitivity to environmental pressure and barriers increases [33]. Hence, one could hypothesize that certain environmental characteristics (e.g., sidewalk evenness) are more important for older adults who suffer from functional limitations or fear from falling. However, such hypotheses have received mixed support [11, 34–36]. Other potentially relevant moderators are psychosocial factors regarding walking for transport (e.g. attitude, self-efficacy, habit), and current walking for transport level [32]. The identification of environmental factors that especially appeal to older adults with a less favorable psychosocial profile (i.e. low attitude, self-efficacy towards walking) or to infrequent walkers, can help to inform environmental interventions regarding how to promote walking for transport among the least active.

Environmental preference may also be influenced by the environmental characteristics of the street in which one lives. For example, older adults residing in a street with heavy traffic may experience this hazard when walking for transport on a daily basis and, therefore, pay more attention to this particular environmental characteristic. Furthermore, older adults with a general preference for nature (and, therefore, also for streets with vegetation while walking for transport) may have self-selected themselves to live in a street with a lot of vegetation. Currently, knowledge regarding the moderators of associations between micro-scale environmental factors and walking for transport is limited [21]. While conjoint analyses do not allow the examination of moderators of relationships between micro-scale environmental factors and appeal for transportation walking directly (by means of interaction effects), it is possible to investigate the existence of subgroups that have different environmental preferences (by means of latent class analysis) [31, 37]. These subgroups can then be compared according to characteristics hypothesized to influence (or moderate) the relationships between the micro-scale environmental factors and a street’s appeal for transportation walking.

The primary aim of the current study was to investigate the perceived influence of a large set of micro-scale environmental factors on a street’s appeal for transportation walking using manipulated photographs of a street among a large sample of older adults. Further aims were to examine whether there were subgroups that differed in environmental preferences for transportation walking, and whether these subgroups differed by socio-demographic, functional and psychosocial characteristics, current level of walking, and environmental perceptions of their own street.

## Methods

### Protocol and participants

A computerized structured questionnaire and a choice-based conjoint exercise with manipulated photographed streets was developed using Sawtooth Software (SSIWebversion 8.3.8). Data were collected through: (1) an online questionnaire; or (2) an interview version of the questionnaire. Interviews were performed to reach older adults who do not have access to or use the Internet. According to the annual study on ICT-use in Belgian households in 2013, 52 % of the Flemish 65 to 74 year-olds did not use the Internet during the last three months [38].

Several sampling strategies were used to recruit Flemish older participants. The online recruitment occurred by contacting (senior) organizations and asking them to disseminate information about the study with a link to the questionnaire among their members. The information and link was posted on their websites, published in their newsletters and/or spread via Facebook or e-mail. Organizations contacted included member organizations of the Flemish Senior Council (including political, socio-cultural and leisure organizations), city and municipal governments, social services and senior councils of cities and municipalities, health funds, organizations providing courses for older adults, and websites specifically targeting older adults. Flyers with information about the study were also distributed via shops, libraries and local service centers. After filling out the online questionnaire, participants were also asked to send the questionnaire link to their relatives.

Recruitment for the interview-administration of the questionnaire occurred via service flat residences and local service centers. A resident of a service flat lives independently, but can, if he or she wishes, make use of services to clean, cook or nurse. Local service centers target people in a novice care situation, are located within the neighborhood and offer informative and recreational activities to stimulate self-reliance. The researchers visited twelve service flat residences (four owned privately and eight owned by the public center for social welfare) and seven local service centers across Flanders. In the service flat residences, our visit was announced by an information letter for each resident, in the social service centers our visit was announced by flyers and posters. During our visit, participants could come to a communal area where we interview-administered the computerized questionnaire.

For inclusion in the study, participants had to be 65 years or older and non-institutionalized. Prior to data collection, the protocol and questionnaires were pilot-tested among ten older adults and questions that were unclear or ambiguous were modified. Actual data collection was performed between November 2014 and January 2015. Completing the questionnaire took approximately 30 min. Informed consent was automatically obtained when participants completed the questionnaire. The study protocol was approved by the ethical committee of the Brussels and Ghent University hospital.

### Development of manipulated photographs

**Fig. 1:**
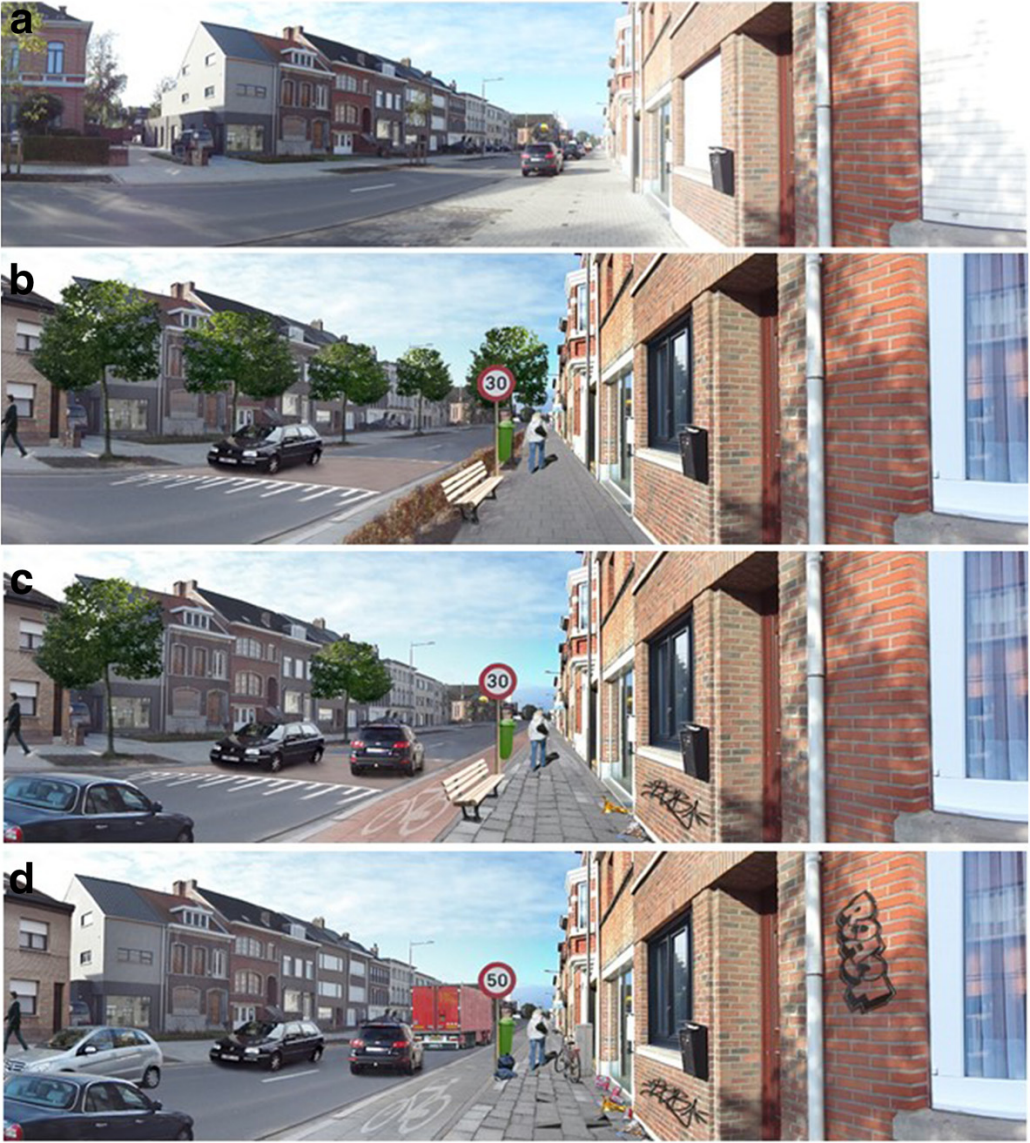
The basic photograph (**a**) and the manipulated best (**b**), medium (**c**) and worst (**d**) street

The panoramic photographs were all modified versions of one “basic” panoramic photograph (see Fig. 1a). This basic photograph was taken at eye level from the sidewalk in a typical (semi-)urban street in Flanders (Belgium). The original photograph itself was not included in the choice-based conjoint exercise, because it was necessary to modify it slightly to be able to perform the intended manipulations. The original photograph was experimentally manipulated on nine environmental attributes using Adobe Photoshop® software. The selection of environmental attributes to be manipulated (see Table 1) was based upon the environmental attributes that appeared to be most important for walking for transport in three previous studies with Flemish older adults [17, 30, 39]. Four environmental attributes had two (e.g. absence vs. presence of a bench) levels and five attributes had three levels (e.g. very uneven, slightly uneven and even sidewalk). Photographs with all possible combinations between the environmental attributes were created, yielding 3888 (= 2^4^ × 3^5^) photographs. Figures 1b and 1d represent the anticipated best and worst street for walking for transport. Figure 1c represents a street with the medium levels of the environmental attributes with three levels (and the anticipated best level for the environmental attributes with two levels).

**Table 1:** Manipulated environmental factors with their levels

Environmental factors	Levels
Sidewalk evenness	Very uneven
	Slightly uneven
	Even
Separation from traffic	No separation
	Cycling path in other color (red)
	Small shrub between sidewalk and cycling path
Obstacle on sidewalk	Obstacle (electrical box and bicycle on sidewalk)
	No obstacle
Traffic volume	4 cars + 1 truck
	3 cars
	1 car
Speed limit	50 km/h
	30 km/h
Traffic calming device	No speed bump
	Speed bump
Overall upkeep	Bad upkeep (a lot of litter and graffiti)
	Moderate upkeep (a bit of litter and graffiti)
	Good upkeep (no litter and graffiti)
Vegetation	No tree
	Two trees
	Five trees
Benches	No bench
	One bench

### Structured questionnaire

The structured questionnaire collected socio-demographic information (i.e., age, gender, area of residence, place of birth, marital status, car ownership, educational level, and former main occupation), functional limitations, use of walking aids, fear of falling, psychosocial factors, current walking for transport level, and environmental perceptions. To obtain information on mode of online recruitment, participants were also asked to indicate which channel they received the link to the questionnaire through.

To assess physical functioning, the physical functioning scale of the validated RAND SF-36 questionnaire was used [40, 41]. Participants were asked to indicate how their health limited their ability to perform ten activities of daily living (e.g. climbing stairs, washing and dressing, etc.) on a 3-point scale: severely, somewhat, or not limited. Following the RAND SF-36 scoring protocol (available on http://www.rand.org/health/surveys_tools/mos/mos_core_36item.html), these responses were recoded as: 0; 50; and 100, respectively, and averaged to obtain the variable ‘physical functioning’ with a higher score indicating greater levels of functioning.

To assess use of a walking aid, participants selected one of the following response options: no; use a cane; or use a walker and/or a wheelchair. These responses were dichotomized into ‘not using a walking aid’ versus ‘using a walking aid’. Fear of falling was measured using the validated Falls Efficacy Scale-International [42, 43]. This scale consists of 16 items (e.g. cleaning the house, getting in or out of a chair, walking on an uneven surface) for which participants indicated how concerned they were about falling when performing these activities on a 4-point scale (1 = not at all concerned, 4 = very concerned).

Questions targeting psychosocial variables were similar to those used in previous studies [44, 45], but applied specifically to walking for transport to a destination within 10 min walking distance. Preference for active/passive transport was assessed by asking ‘which transport mode do you prefer to travel to a destination located within a 10 min walking distance?’: car; motorbike; public transport; bicycle; electrical bicycle; walking; or scooter. Self-efficacy was assessed with a single item: ‘How confident are you that you can continue to walk to a destination located within a 10 min walking distance when conditions are difficult (e.g. bad weather, you feel tired, etc.)?’. Response options ranged from (1) ‘I am sure I cannot continue with walking’ to (5) ‘I am sure I can continue with walking’.

Social support was assessed with a single item: ‘Do you receive support from family and friends to walk to a destination located within a 10 min walking distance?’. Perceived benefits (‘Do you believe that walking to a destination located within a 10 min walking distance has many benefits for you (e.g. fresh air, pleasant, healthy, etc.)?’) and barriers (‘Do you experience many barriers to walk to a destination located within a 10 min walking distance (e.g. lack of time, health problems, bad weather, etc.)?’) were assessed with two items. A single item assessed intention to walk by asking: ‘Imagine that you live in a neighborhood where it is easier to walk to a destination located within a 10 min walking distance (e.g. there are more shops nearby, it is safer to walk…). Do you think you would walk more to destinations located within a 10 min walking distance?’ For the latter four constructs, response options ranged from: (1) ‘no, definitely not’ to (5) ‘yes, definitely’. Habit was assessed by four items that asked whether walking for transport is something: (1) they automatically do; (2) that belongs to their daily routine; (3) that typifies them; (4) that they do for a long time already. Response options ranged from: (1) completely disagree to (5) completely agree. Responses on the four items were averaged to obtain the variable ‘habit’.

To assess current walking for transport and walking for recreation levels, a section of the validated International Physical Activity Questionnaire (IPAQ, long form, last 7 days) was used [46]. Participants were asked to report the frequency of walking for transport during the last seven days and the average duration of walking for transport on one of those days. Weekly minutes of walking for transport was calculated by multiplying the reported number of days by the duration of walking for transport on one of those days (standard scoring procedures available on http://www.ipaq.ki.se/). Weekly minutes of walking for recreation was assessed and calculated similarly.

**Fig. 2:**
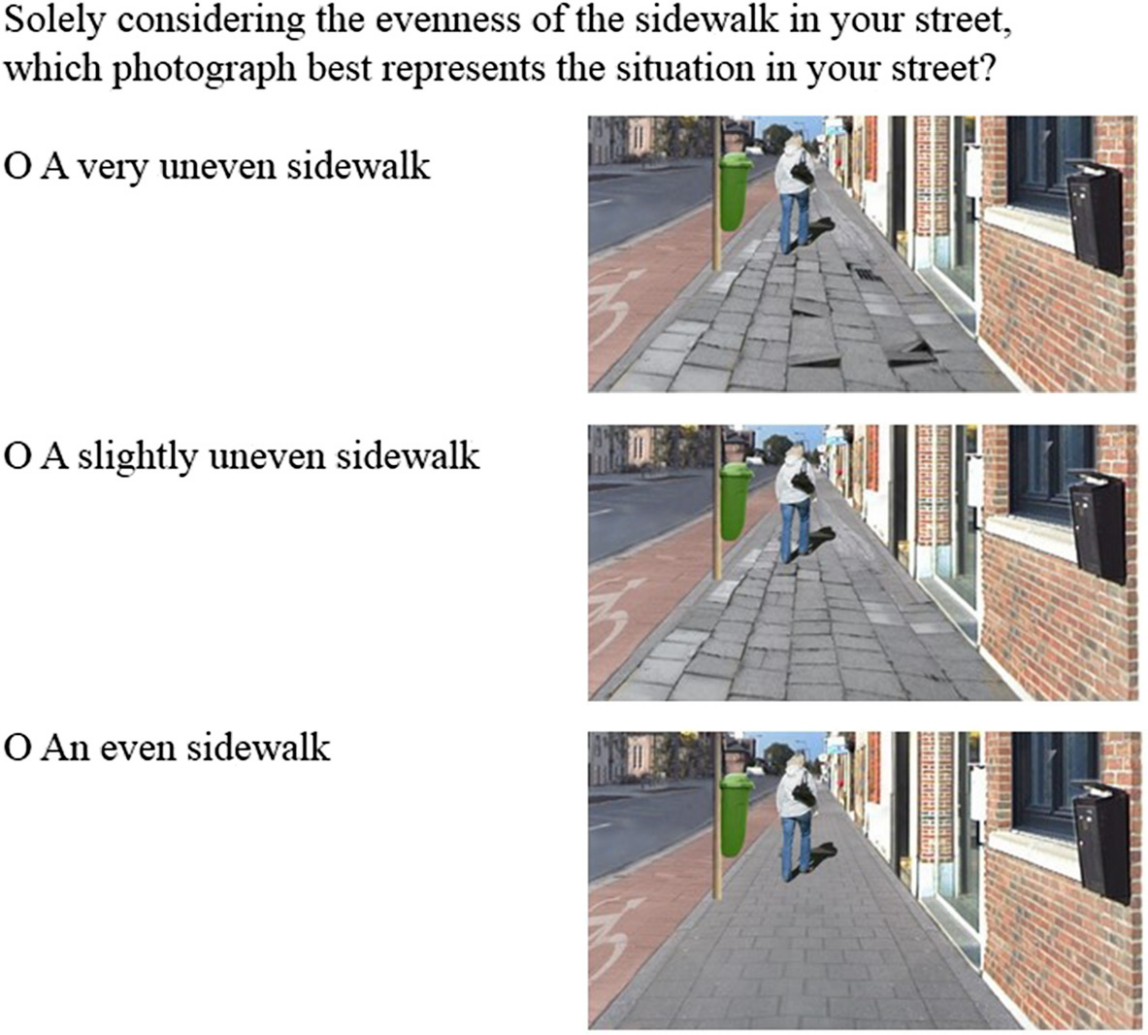
Example of how environmental perceptions were assessed

Environmental perceptions of participants’ own street were assessed by asking how participants perceived their street in terms of sidewalk evenness, separation from traffic, sidewalk width, traffic volume, speed limit, traffic calming devices, overall upkeep, presence of vegetation and presence of benches. These environmental factors corresponded to the factors that were manipulated in the photographs. Similarly, the response options corresponded to the levels of the manipulated factors in the photographs and the manipulated photographs were used to illustrate the response options. An example of the question assessing perceived sidewalk evenness is provided in Fig. 2 (assessment of the eight remaining environmental perceptions is available in Additional file 1). Since these questions were especially developed for the current study, test-retest reliability was assessed in a subsample (*n* = 46). All questions had substantial to perfect test-retest reliability (kappa’s > 0.60), except for separation sidewalk-cycling path (kappa = 0.51) and overall upkeep (kappa = 0.45), which had moderate reliability [47].

### Choice-based conjoint exercise

To investigate the effects of the environmental manipulations on the appeal of a street for transportation walking a choice-based conjoint exercise was developed using Sawtooth Software (SSIWeb version 8.3.8). Choice-based conjoint exercises are frequently used in marketing research to study consumer preferences. During a choice-based conjoint exercise, participants are asked to choose between products (e.g. televisions) that differ in some pre-defined characteristics (e.g. screen size, screen quality and price) (Orme, 2009). In the current study the ‘products’ are streets that differed in nine pre-defined environmental attributes. This methodology has been used previously to study older adults’ preferences for public open spaces [48] and walking programs [49]. While these previous studies used text descriptions, the current study used the manipulated photographs to depict the environmental attributes of the streets. Responses to color photographs have been shown to accurately reflect on-site responses to real environments [50, 51].

The choice-based conjoint exercise consisted of 20 choice tasks in which participants were required to choose between two manipulated streets. Participants were requested to indicate which street they would prefer for transportation walking. The choice task was full-profile, which implies that the two streets presented in one task could differ in a minimum of one and a maximum of nine environmental attributes (Orme, 2009). The conjoint exercise started with three training tasks to get acquainted with the format; these three exercises were similar for all participants and were not used in the analyses. The remaining 17 tasks consisted of 14 random and three fixed tasks. The 14 random tasks were different for all participants and were randomly assigned by the software using the recommended balanced overlap method [52]. The three fixed tasks were similar for all participants and two of these tasks were exactly the same to enable examination of test-retest reliability. The results of the fixed tasks were also compared against the predictions of the final statistical model to check the validity of the statistical model.

Prior to the choice-based conjoint exercise participants received the following standardized instructions: “Imagine yourself walking to a friend’s home located 10 min from your home during daytime. The weather is ideal for walking, it is not too warm, not too cold, there is no wind, and it is not raining. Two photographs of streets will be presented to you, one in the upper part of the screen and another one in the lower part of the screen. Each photograph depicts the same street, but you will notice that certain things differ from photograph to photograph. The purpose is that you indicate which street you would choose to walk along. The distance to your friend’s home is the same along both streets. There is no good or bad solution, we are just interested in what you consider as most important while walking to your friend’s home.” By instructing participants to imagine walking to a friend’s home located ten minutes from their own home, a specific context was provided [53] and distance to the destination was standardized.

### Data analyses

Online recruitment yielded 1442 eligible participants filling out the questionnaire. Participants not completing the choice task (*n* = 412) were removed from the dataset resulting in 1030 complete cases. Additionally, 169 older adults completed an interview-administered version of the questionnaire yielding a total sample size of 1199 participants. Test-retest reliability of the two fixed tasks resulted in a percentage agreement of 94.3 % which corresponds to 68 participants not responding consistently. These inconsistent responders were removed yielding a final analytic sample of 1131 participants. The inconsistent responders were older, more likely to live in a service flat and had the questionnaire administered by interview than consistent responders.

Descriptive characteristics of the sample were calculated using SPSS Statistics 22. Data obtained from the choice-based conjoint exercise were analyzed using Sawtooth Software SSI Web version 8.3.8. Choice-based conjoint analyses yield two types of parameters: part-worth utilities and importances [31]. A part-worth utility of an environmental attribute level can be interpreted similar to a regression coefficient and represents the desirability of the environmental attribute level. For ease of interpretation, these part-worth utilities were zero-centered. For example, if the attribute levels ‘very uneven’, ‘slightly uneven’ and ‘even’ sidewalk have part-worth utilities -5.0, 1.0 and 4.0, respectively, this means that a very uneven sidewalk is the least preferred and an even sidewalk is the most preferred level. Importances reflect the impact an environmental attribute has on choice (with greater importances reflecting greater impacts on choice). It should be noted that importances are directly related to the environmental attribute ranges (i.e. the difference between the least and most favorable environmental attribute level) that were used in the experiment.

The conjoint analyses were performed in three steps. First, part worth utilities and importances were calculated in the total sample using Hierarchical Bayes estimation as recommended [31, 54]. Administration mode (individually online versus interview) was entered as a covariate in the analyses. Preliminary iterations were run until convergence was reached and, consecutively, 10,000 draws were used per respondent. Average part-worth utilities and importances were calculated and 95 % confidence intervals were constructed to compare part-worth utilities and importances. Part-worth utilities within one attribute and importances with non-overlapping 95 % confidence intervals are significantly different from each other with alpha = 0.05. The fit of the conjoint model was presented by the Root LikeliHood (RLH) which ranges between 0 and 1. For a choice exercise with two alternatives, the RLH should be substantially larger than 0.50 (the predictability of the response using uninformative utilities) [31]. Furthermore, to assess the validity of the models, we presented the percentage of agreement between the choice predicted by the model and the actual choice of the participants in the two different fixed tasks. This represents for how many participants the choice predicted by the model corresponded to the actual choice of the participants.

In a second step, latent class analysis with 15 replications was performed to examine whether subgroups of the population could be identified that have different environmental preferences for transportation walking [37]. Based on increases in model fit and number of participants in each subgroup, a four-subgroup solution was selected. Since it is not possible to enter covariates in latent class analyses, we also performed latent class analyses in a subsample including only participants that completed the questionnaire individually online. This yielded similar results. To obtain part-worth utilities and importances in the four subgroups, hierarchical Bayes estimation was performed separately in each subgroup following the same procedures as described in the first step. Using the Advanced Test design of Sawtooth Software SSI Web we performed an a priori power analysis by means of simulation. To obtain equivalent standard errors within each attribute and sufficiently precise estimates (defined as standard errors < 0.05), the power analyses showed that each subgroup should include 90 participants [52].

In a third step, we used SPSS statistics 22 to examine whether the four subgroups obtained in step two differed in socio-demographic, functional and psychosocial characteristics, current level of walking and environmental perceptions of their own street. For continuous variables, differences between the four subgroups were examined using MANOVAs, we interpreted Wilks’ Lambda’s with Tukey post-hoc analyses when variances were homogenous and Tamhane post-hoc analyses when variances were heterogeneous [55]. For categorical variables, differences between the four subgroups were examined using chi square tests. Significance level was defined at alpha = 0.05.

## Results

From the analytic sample of 1131 participants, 83.1 % completed the questionnaire online. The most important channels from which these participants received the link to the questionnaire were: relatives (24.3 %), senior organizations (13.0 %), websites or newsletters from their health funds (10.6 %), city/municipalities (7.8 %) and the Flemish senior council (7.0 %), other websites (12.9 %), and city/municipal senior councils (8.5 %).

### Descriptive characteristics of the sample

Table 2 presents the socio-demographic, functional and psychosocial characteristics, and current walking levels of the sample. Table 3 presents participants’ perceptions of their own street. About 74 % of the participants reported having a sidewalk in their street. Of the participants with a sidewalk in their street, 54 % reported the sidewalk to be very (12 %) or slightly uneven (42 %). The most prevalent street configuration was a street without a cycling path and the sidewalk being separated from traffic by a curb (59 %).

**Table 2:** descriptive characteristics of the sample (*n* = 1131)

Age (M ± SD)	71.9 ± 6.2
Gender (% women)	47.5
Country of birth (% born in Belgium)	95.6
Educational level (% with tertiary education)	36.3
Former main occupation (%)	
Household	9.5
Blue collar	22.8
White collar	67.7
Marital state	
Married/co-habiting	66.6
Widowed	20.7
Divorced	7.6
Single and never been married	5.1
Area of residence (% rural)	59.1
Residential situation (% in service flat)	12.0
BMI (kg/m^b^, M ± SD)	26.5 ± 4.0
Physical functioning (/100, M ± SD)^a^	83.3 ± 21.4
Use of walking aid (%)	12.6
Fear of falling (/4, M ± SD)^b^	1.3 ± 0.5
Transport preference (%)	
By foot	42.1
Bicycling	34.2
Motorized transport^c^	23.6
Habit (/4, M ± SD)	2.9 ± 1.4
Self-efficacy (/5, M ± SD)	3.5 ± 1.5
Social support (/5, M ± SD)	2.7 ± 1.5
Perceived benefits (/5, M ± SD)	4.3 ± 1.0
Perceived barriers (/5, M ± SD)	2.5 ± 1.3
Intention (/5, M ± SD)	3.8 ± 1.3
Walking for transport (min/week, M ± SD)	125.7 ± 156.5
Walking for recreation (min/week, M ± SD)	145.2 ± 204.6

*M* mean, *SD* standard deviation

^a^scale with 0 = minimum physical functioning and 100 = maximum physical functioning

^b^scale with 1 = minimum fear from falling and 100 = maximum fear from falling

^c^including public transit (4.3 %)

**Table 3:** Participants’ environmental perceptions of their own street

Sidewalk presence (%)	74.1
Sidewalk evenness (%)^a^	
Very uneven	12.1
Slightly uneven	42.1
Even	45.8
Separation from traffic (%)^a^	
No cycling path, separation from traffic by a curb	59.1
No cycling path, real separation from traffic (parked cars, shrub…)	13.5
Sidewalk separated from cycling path by a curb	10.0
Sidewalk separated from cycling path by color	10.4
Sidewalk separated from cycling path by real separation (parked cars, shrub…)	7.0
Obstacle (% without obstacle on the sidewalk)^a^	55.8
Traffic volume (%)	
Heavy traffic	25.6
Medium traffic	37.7
Light traffic	36.7
Speed limit (%)	
90 km/h	0.9
70 km/h	8.7
50 km/h	62.0
30 km/h	28.5
Presence of traffic calming (% with traffic calming)	30.9
Overall upkeep (%)	
Not clean at all	7.5
Moderately clean	29.6
Very clean	62.9
Vegetation (%)	
No vegetation	18.7
Some vegetation	32.7
A lot of vegetation	48.6
Presence of bench (% with bench)	17.5

^a^Percentages calculated for streets where a sidewalk is present

### Environmental preference for transportation walking in the total sample

**Fig. 3:**
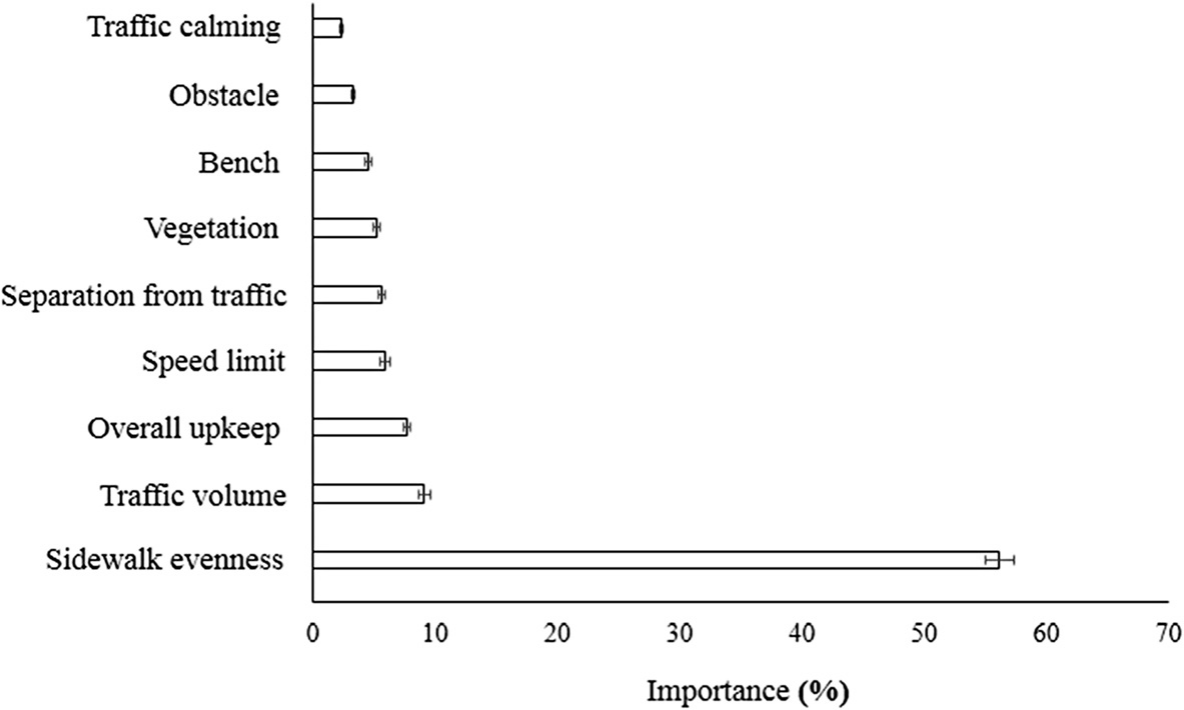
Sidewalk evenness was the most important environmental attribute in the total sample

In the total sample, sidewalk evenness was the most important street feature for transportation walking (56.2 %; 95 % CI = 55.0, 57.4), followed by traffic volume (9.1 %; 95 % CI = 8.6, 9.6) and overall upkeep (7.7 %; 95 % CI = 7.5, 7.9) (see Fig. 3). These were followed by speed limit (5.9 %; 95 % CI = 5.6, 6.3) and separation from traffic (5.7 %; 95 % CI = 5.4, 6.0) for which the importances did not significantly differ from each other. The importance of vegetation (5.2 %; 95 % CI = 4.9, 5.5) was significantly lower than speed limit, but not than separation from traffic. Consecutively, importances decreased significantly for the presence of a bench (4.5 %; 95 % CI = 4.2, 4.8), an obstacle on the sidewalk (3.3 %; 95 % CI = 3.2, 3.4) and traffic calming (2.3 %; 95 % CI = 2.2, 2.5).

Within each environmental attribute part-worth utilities significantly increased in the expected direction (see Table 4, first column). For example, within sidewalk evenness, a very uneven sidewalk had a significantly lower part-worth utility (-267.2; 95 % CI = -273.2, -261.1) than a slightly uneven sidewalk (36.9; 95 % CI = 35.6, 38.1) which again had a significantly lower part-worth utility than an even sidewalk (230.3; 95 % CI = 224.5, 236.2). This implies that an even sidewalk was preferred over a slightly uneven sidewalk which in turn was preferred over a very uneven sidewalk.

**Table 4:** Part-worth utilities of the environmental attribute levels in the total sample and the four subgroups

Environmental factors	Total sample (*n* = 1131)	Subgroup 1 (*n* = 624)	Subgroup 2 (*n* = 350)	Subgroup 3 (*n* = 83)	Subgroup 4 (*n* = 74)
	Part-worth utility (95 % CI)	Part-worth utility (95 % CI)	Part-worth utility (95 % CI)	Part-worth utility (95 % CI)	Part-worth utility (95 % CI)
Sidewalk evenness					
Very uneven	-267.2 (-273.2, -261.1)	-332.5 (-334, -331)	-228.4 (-231.9, -224.8)	-40.1 (-44.2, -36.1)	-29.9 (-39.2, -20.6)
Slightly uneven	36.9 (35.6, 38.1)	16.7 (15.9, 17.6)	58.2 (54.9, 61.6)	23.7 (16.7, 30.6)^a^	10.1 (0.3, 19.8)^a^
Even	230.3 (224.5, 236.2)	315.8 (314.1, 317.5)	170.1 (167.7, 172.5)	16.5 (9.8, 23.1)^a^	19.8 (11.6, 28)^a^
Separation from traffic					
No separation	-11.9 (-13.3, -10.4)	-12.1 (-12.7, -11.5)	-11.4 (-15.1, -7.7)	4.5 (-0.7, 9.7)^a^	-17.3 (-27.5, -7.1)^a^
Cycling path in other color (red)	0.6 (-0.6, 1.8)	1 (0.4, 1.6)	-1.6 (-4.8, 1.6)	6.1 (2.3, 9.9)^a^	-19.7 (-30, -9.3)^a^
Small shrub between sidewalk and cycling path	11.3 (9.2, 13.3)	11.1 (10.2, 12)	13 (8.4, 17.6)	-10.6 (-15.7, -5.5)	37 (22.9, 51.1)
Presence of obstacle					
Electrical box and bicycle on sidewalk	-12.4 (-13.1, -11.7)	-15.7 (-16.2, -15.2)	-11 (-12.9, -9.2)	-14.9 (-20.3, -9.6)	-1.2 (-8, 5.7)^a^
No obstacle present	12.4 (11.7, 13.1)	15.7 (15.2, 16.2)	11 (9.2, 12.9)	14.9 (9.6, 20.3)	1.2 (-5.7, 8)^a^
Traffic volume					
4 cars + 1 truck	-38.5 (-41.4, -35.6)	-11.6 (-12.6, -10.6)	-58.9 (-62.8, -55)	-168.4 (-187.1, -149.8)	-16.1 (-23.7, -8.4)
3 cars	7.7 (6.6, 8.9)	1.2 (0.4, 1.9)	16.8 (14.2, 19.4)	30.4 (20.7, 40.1)	4.4 (-3.3, 12.1)^a^
1 car	30.8 (28.3, 33.2)	10.4 (9.6, 11.2)	42.1 (38.2, 46)	138 (126.6, 149.5)	11.6 (3.6, 19.7)^a^
Speed limit					
50 km/h	-18.5 (-20.4, -16.5)	-14.6 (-15.3, -14)	-19 (-22.1, -15.8)	-78.8 (-93.1, -64.6)	18.1 (3.2, 33)
30 km/h	18.5 (16.5, 20.4)	14.6 (14, 15.3)	19 (15.8, 22.1)	78.8 (64.6, 93.1)	-18.1 (-33, -3.2)
Traffic calming device					
No speed bump	-4.4 (-5.3, -3.4)	-3.1 (-3.7, -2.5)	-1.8 (-3.7, 0.2)^a^	-26.8 (-35.1, -18.6)	-5.8 (-15.6, 4)^a^
Speed bump present	4.4 (3.4, 5.3)	3.1 (2.5, 3.7)	1.8 (-0.2, 3.7)^a^	26.8 (18.6, 35.1)	5.8 (-4, 15.6)^a^
Overall upkeep					
Bad upkeep (a lot of litter and graffiti)	-25.7 (-27.2, -24.3)	-32.2 (-33, -31.4)	-28.3 (-31.7, -25)	-29.7 (-35.1, -24.3)	19.9 (9.5, 30.4)
Moderate upkeep (a bit of litter and graffiti)	-3 (-3.9, -2)	-4.6 (-5.3, -3.9)	-0.4 (-2.4, 1.6)	7.6 (1.6, 13.6)	-15.2 (-22.4, -8)^a^
Good upkeep (no litter and graffiti)	28.7 (27.1, 30.3)	36.8 (35.8, 37.9)	28.7 (25.3, 32.2)	22.1 (16.9, 27.3)	-4.7 (-15.9, 6.4)^a^
Vegetation					
No tree	-11.1 (-12.7, -9.4)	1.4 (0.8, 2.1)	-18.9 (-22.5, -15.3)	-39.5 (-45.5, -33.5)	-69.2 (-83.2, -55.2)
Two trees	-5.6 (-6.5, -4.7)	-4.3 (-5.1, -3.4)	-8.3 (-10.5, -6.2)	9.6 (3.6, 15.6)	3.7 (-6.8, 14.3)
Five trees	16.7 (15, 18.4)	2.8 (1.9, 3.7)	27.2 (23.8, 30.6)	29.9 (25.1, 34.7)	65.4 (46.6, 84.3)
Presence of bench					
No bench	-15.5 (-17, -14)	-14.7 (-15.4, -13.9)	-11.4 (-13.7, -9)	-6.1 (-9.2, -3)	-68.2 (-83.8, -52.6)
Bench present	15.5 (14, 17)	14.7 (13.9, 15.4)	11.4 (9, 13.7)	6.1 (3, 9.2)	68.2 (52.6, 83.8)
RLH	0.92	0.98	0.88	0.93	0.86
Agreement model prediction - fixed task 1 (%)^b^	82.2	93.8	58.3	79.5	75.7
Agreement model prediction - fixed task 2 (%)^b^	97.5	100.0	99.1	91.6	78.4

Part-worth utilities should be compared within one environmental factor and one subgroup (not across factors and subgroups)

^a^Within one environmental factor and one subgroup, levels with an “ ^a^ ” do not differ significantly

^b^This represents for how many participants the choice predicted by the model corresponds to the actual choice of the participants

*CI* confidence interval, *RLH* root likelihood

### Subgroups differing in environmental preferences for transportation walking

**Fig. 4:**
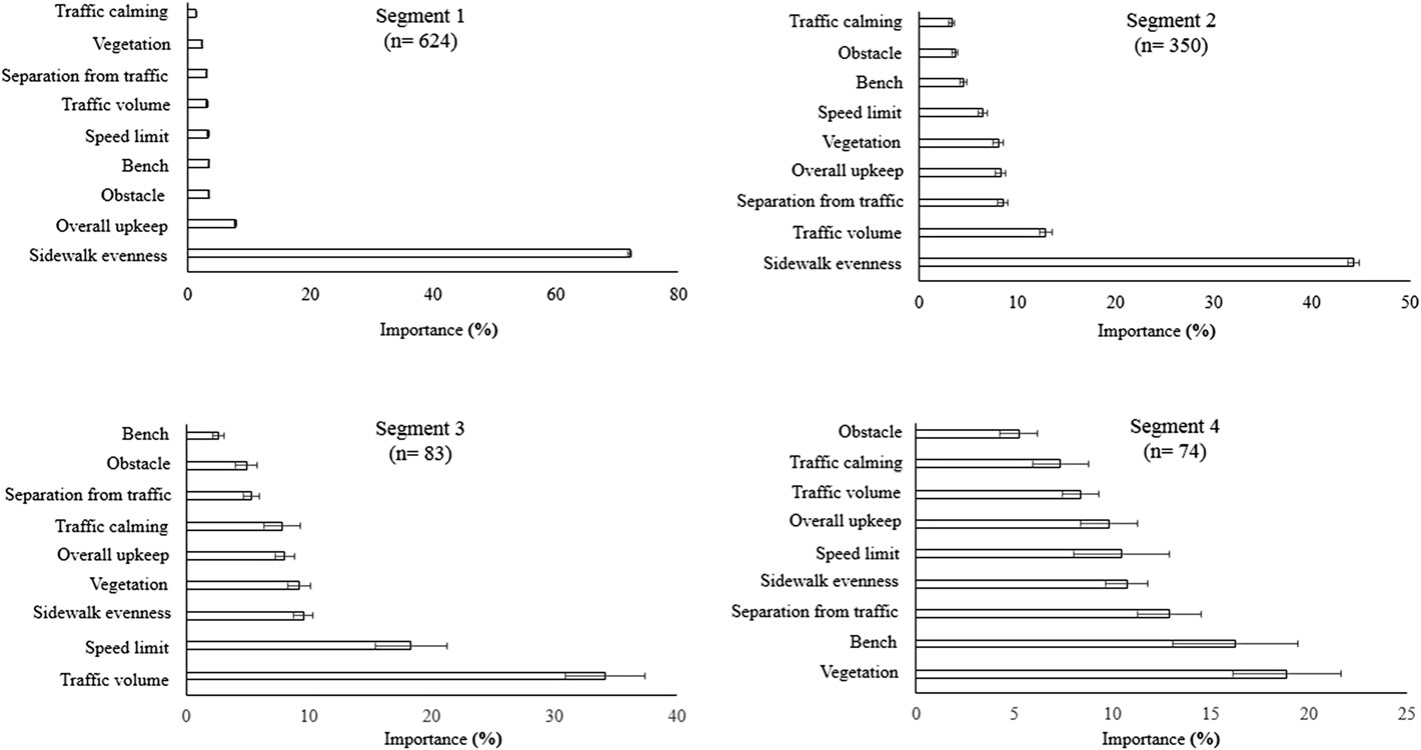
Importances of the environmental attributes in the four subgroups

Latent class analyses revealed four subgroups differing in environmental preferences for transportation walking. The importances of the environmental attributes within the four subgroups are presented in Fig. 4 and the corresponding part worth utilities are presented in Table 4.

Subgroup 1 was the largest representing 624 participants (55.2 % of the total sample). For this subgroup, with an importance of 72.0 % (95 % CI = 71.7, 72.4), sidewalk evenness was by far the most important environmental attribute influencing preference for transportation walking. Importances of the other environmental attributes were much lower. For example, the second most important attribute, overall upkeep, had an importance of only 7.7 % (95 % CI = 7.5, 7.9). The importances of the presence of an obstacle on the sidewalk (3.5 %; 95 % CI = 3.4, 3.6), presence of a bench (3.5 %; 95 % CI = 3.3, 3.6), and speed limit (3.3 %; 95 % CI = 3.2, 3.5) were significantly lower than overall upkeep, but did not differ significantly from each other. The importance of traffic volume (3.1 %; 95 % CI = 3.0, 3.3) was significantly lower than the importance of the presence of an obstacle and bench, but not than the importance of speed limit. The importance of separation from traffic (3.1 %; 95 % CI = 3.0, 3.2) was significantly lower than the importance of the presence of an obstacle, bench, and speed limit, but not than the importance of traffic volume. The importances further significantly decreased for vegetation (2.4 %; 95 % CI = 2.2, 2.5) and traffic calming (1.3 %; 95 % CI = 1.3, 1.4). Similar to our findings in the total sample, part-worth utilities significantly increased in the expected direction within each environmental attribute (see Table 4).

Subgroup 2 included 350 participants (30.9 % of the total sample) for which, similar to subgroup 1 although less pronounced, sidewalk evenness was the most important environmental attribute (44.3 %; 95 % CI = 43.7, 44.8). The second most important attribute was traffic volume (12.9 %; 95 % CI = 12.2, 13.5). Separation from traffic (8.5 %; 95 % CI = 8.0, 9.1), overall upkeep (8.3 %; 95 % CI = 7.8, 9.1) and vegetation (8.0 %; 95 % CI = 7.6, 8.5) had a significantly lower importance than traffic volume, but their importances did not significantly differ from each other. Speed limit had a significantly lower importance (6.5 %; 95 % CI = 6.0, 7.0) than separation from traffic, overall upkeep and vegetation, but had a significantly higher importance than presence of a bench (4.5 %; 95 % CI = 4.1, 4.8). Presence of a bench had a significantly higher importance than presence of an obstacle (3.7 %; 95 % CI = 3.4, 4.0) and traffic calming (3.3 %; 95 % CI = 3.0, 3.6). The importances of these two latter attributes did not differ significantly. For subgroup 2, within each environmental attribute part-worth utilities significantly increased in the expected direction, except for traffic calming device (see Table 4). No significant difference in preference for absence or presence of a traffic calming device was observed.

In subgroup 3, which included 83 participants (7.3 % of the total sample), the highest importance was observed for traffic volume (34.2 %; 95 % CI = 30.9, 37.4), followed by speed limit (18.3 %; 95 % CI = 15.4, 21.3), sidewalk evenness (9.5 %; 95 % CI = 8.7, 10.3), vegetation (9.2 %; 95 % CI = 8.3, 10.2), overall upkeep (8.0 %; 95 % CI = 7.3, 8.8), and traffic calming (7.8 %; 95 % CI = 6.3, 9.3). The importances of the four latter attributes did not differ significantly from each other. Importances further decreased for separation from traffic (5.3 %; 95 % CI = 4.7, 5.9) and presence of an obstacle on the sidewalk (4.9 %; 95 % CI = 4.0, 5.8). The importances of these two attributes did not significantly differ from each other. Presence of a bench had the lowest importance (2.7 %; 95 % CI = 2.2, 3.1). Within each environmental attribute for subgroup 3, part-worth utilities significantly increased in the expected direction, except for sidewalk evenness and separation from traffic (see Table 4).

In subgroup 4, which included 74 participants (6.5 % of the total sample), differences in importances between the environmental attributes were less pronounced. The highest importances were found for vegetation (18.9 %; 95 % CI = 16.2, 21.6) and presence of a bench (16.2 %; 95 % CI = 13.1, 19.4), which did not differ significantly from each other. Separation from traffic (12.9 %; 95 % CI = 11.3, 14.5) had a significantly lower importance than vegetation but not than presence of a bench. Sidewalk evenness (10.7 %; 95 % CI = 9.7, 11.8) and traffic calming (10.5 %; 95 % CI = 8.0, 12.9) had significantly lower importances than vegetation and presence of a bench but not than separation from traffic. Overall upkeep (9.8 %; 95 % CI = 8.4, 11.3) had a significantly lower importance than separation from traffic but not than sidewalk evenness and traffic calming. Importances for traffic volume (8.4 %; 95 % CI = 7.4, 9.3) and traffic calming device (7.4 %; 95 % CI = 5.9, 8.8) were not significantly lower than traffic calming device and overall upkeep. Presence of an obstacle (5.2 %; 95 % CI = 4.3, 6.2) had the lowest importance but did not differ significantly from traffic calming device. In subgroup 4, only two attributes followed the expected direction in terms of increases in part-worth utilities; vegetation and presence of a bench. The part-worth utilities of the remaining seven attributes did not follow the anticipated direction (see Table 4).

### Differences between the subgroups in socio-demographic, functional, psychosocial, walking and environmental variables

Table 5 presents the differences in socio-demographic, functional, psychosocial, walking and environmental variables between the four subgroups. Subgroup 1 contained a high percentage of women (51.9 %), and a low percentage of participants residing in a service flat (11.1 %), walking aid use (11.4 %) and presence of traffic calming in their own street (27.6 %) compared to the other subgroups. Participants in subgroup 1 also reported more perceived barriers compared to participants in subgroup 2 (p = 0.03).

**Table 5:** Differences in socio-demographic, functional, psychosocial, walking and environmental variables between the subgroups

	Subgroup 1 (*n* = 624)	Subgroup 2 (*n* = 350)	Subgroup 3 (*n* = 83)	Subgroup 4 (*n* = 74)	Chi^2^ (p-value)	Wilks’ lambda F (p-value)^d^
Gender (% women)	51.9	42.0	37.3	47.3	12.6 (0.01)	
Area of residence (% rural)	57.9	60.6	48.2	74.3	11.9 (0.01)	
Residential situation (% in service flat)	11.1	11.1	13.3	23.0	9.3 (0.03)	
Physical functioning (/100, M ± SD)^1^	83.5 ± 20.4^a,b^	85.2 ± 20.2^a^	81.8 ± 24.4^a,b^	75.5 ± 28.7^b^		4.3 (0.01)
Use of walking aid (%)	11.4	11.1	14.5	28.4	18.5 (<0.001)	
Fear of falling (/4, M ± SD)^2^	1.3 ± 0.5^a,b^	1.3 ± 0.4^a^	1.4 ± 0.5^a,b^	1.6 ± 0.7^b^		5.9 (0.001)
Perceived barriers (/5, M ± SD)	2.6 ± 1.3^a^	2.4 ± 1.2^b,c^	2.2 ± 1.2^a,c^	2.6 ± 1.3^a,b,c^		3.9 (0.01)
Traffic volume in own street (%)						
Heavy traffic	24.4	29.7	19.3	24.3	12.2 (0.06)	
Medium traffic	40.7	32.3	33.7	41.9		
Light traffic	34.9	38.0	47.0	33.8		
Traffic calming in own street (% with traffic calming)	27.6	35.7	31.3	35.1	7.7 (0.05)	
Presence of bench in own street (% with bench)	16.7	16.6	15.7	31.1	10.2 (0.02)	

^a,b,c^Means with different superscripts differ significantly from each other

^d^The multivariate Wilks’ lambda F = 1.5 with p = 0.02

Subgroup 2 contained a low percentage of service flat residents (11.1 %) and walking aid users (11.1), but a high percentage of participants with heavy traffic (29.7) and traffic calming devices in their street (35.7 %). Participants in subgroup 2 had a significantly higher level of functional fitness (p = 0.04) and a lower level of fear of falling (p = 0.02) compared to participants in subgroup 4.

Subgroup 3 included the lowest percentage of women, rural participants and participants reporting heavy traffic in their street. Subgroup 4 included the highest percentage of rural participants, service flat residents, users of walking aids and participants reporting light traffic in their street. It also included the highest percentage of participants reporting the presence of a bench in their street. Furthermore, participants in subgroup 4 were less functionally fit and more fearful of falling than participants in subgroup 2.

The four subgroups did not significantly differ in age, educational level, former main occupation, marital state, BMI, transport preference, habit, self-efficacy, social support, perceived benefits, intentions, or walking for transport or recreation. Their perceptions of sidewalk evenness, separation from traffic, obstacles, speed limit, overall upkeep and vegetation in their own street also did not differ.

## Discussion

The current study aimed to examine the effects of hypothetical changes in micro-scale environmental factors on a street’s appeal for transportation walking among older adults. Our manipulation of sidewalk evenness was given by far the greatest rating of importance of a street’s appeal for transportation walking in the total sample. Furthermore, it was the most important factor in the two largest subgroups representing 86 % of our sample. This is in line with findings from previous qualitative studies in which sidewalk quality emerged as a critical factor influencing walking for transport among older adults [17–20]. In a previous pilot study using manipulated panoramic photographs among Flemish older adults, sidewalk evenness also appeared as the most important environmental factor influencing a street’s appeal for transportation walking [30]. In that pilot study, qualitative data showed that the older participants were afraid of falling and being injured when walking on uneven sidewalks.

Previous quantitative studies have mostly focused on sidewalk availability or used overall measures such as ‘infrastructure and safety for walking’ including items such as ‘presence of sidewalks’, ‘maintenance of sidewalks’, ‘separation from motorized traffic’ and ‘presence of street lighting’ [56]. These studies yielded inconsistent relationships with transportation walking [57–60]. This may be explained by the presence of a sidewalk not stimulating transportation walking when the sidewalk is (very) uneven. Furthermore, while it is possible for observational studies to capture the overall quality of sidewalk infrastructure, they may also obscure the relationship of one environmental factor that strongly relates to transportation walking. For example, a neighborhood may have sidewalks that are well-lit and separated from traffic and, therefore, score relatively high on ‘infrastructure and safety for walking’, but this may not relate to transportation walking if the sidewalks are uneven. Our findings suggest that sidewalk evenness may be a key factor influencing older adults’ preferences for transportation walking. However, it has to be acknowledged that importances obtained from conjoint analysis are a function of the difference between the least and most favorable level of the environmental attribute. It could be argued that our least favorable level of sidewalk evenness was rather extreme. However, 12 % of participants who reported having a sidewalk in their street perceived it to be very uneven. This assessment of perceived sidewalk evenness in their own street was illustrated with the same manipulated photograph of a very uneven sidewalk as used in the choice-based conjoint exercise. Hence, for the vast majority of our sample, sidewalk evenness was the most important environmental attribute and, 12 and 42 % of participants reported their own sidewalk to be very or slightly uneven respectively. Therefore, the provision of even sidewalks can be considered a priority when designing or modifying environments to promote transportation walking among older adults.

A second aim of our study was to examine whether there subgroups exist that differ in their environmental preferences for transportation walking and whether these subgroups can be characterized based on their socio-demographic, functional and psychosocial characteristics, current level of walking and environmental perceptions of their own street. Four subgroups emerged from our analysis. Two subgroups, including 55 and 31 % of all participants, had a clear preference for streets with an even sidewalk. A third and smaller subgroup, including 7 % of the sample, based their choices predominantly on traffic volume (34 %) and speed limit (18 %). This subgroup had the highest percentage of men, urban participants and participants residing in streets with light traffic. Possibly, these participants have a general preference for traffic-calm streets, self-selected themselves to live in streets with light traffic and also prefer streets with light traffic to walk for transport. This implies that in order to avoid discouraging older adults who live in streets with light traffic from walking for transportation, traffic volumes in their streets should not increase (e.g. by limiting cut-through traffic). Traffic volume and speed limit may influence traffic safety. Perceived traffic-related safety has been found to be unrelated to walking for transport among US [60] and Hong Kong older adults [57]. In a sample of Flemish older adults feelings of traffic safety were even negatively associated to the odds of transportation walking [12]. In a study among Dutch older adults, higher levels of objectively measured traffic volume were related to higher use of a street for transportation walking [61]. These previous and current findings seem to suggest that while older adults may prefer to walk in streets with little traffic, they may be forced to walk in streets with heavy traffic to reach their daily destinations. Furthermore, our current findings suggest that traffic volume and speed limit may only be a key factor influencing transportation walking for a limited proportion of older adults (and especially among urban men living in streets with light traffic).

Compared to the other subgroups, the importances of the different environmental attributes in subgroup four, including 7 % of our sample, were more similar. Participants in subgroup four paid most attention to the presence of vegetation (19 %) and a bench (16 %). The importance of the presence of vegetation is somewhat surprising since vegetation and other aesthetic environmental qualities are typically considered to be related to recreational rather than transportation walking [9]. One possible explanation for the importance of vegetation is that subgroup 4 included the highest percentage of service flat residents. In the current sample, service flat residents reported being less functionally fit and walked less for recreation and transportation than participants not residing in service flats (data not shown). Therefore, they can be expected to spend a large amount of their time indoors, which may increase their need for contact with nature and, hence, increase their preference for vegetation in streets [62]. In a sample of Australian retirement village residents, perceived aesthetics (including the item ‘lots of greenery’) of the village environment was related to recreational walking, but not to transportation walking [63]. The ‘greening’ of streets is a relatively low cost feasible environmental modification that local councils could implement to promote walking for transport among those similar to participants in subgroup 4.

The importance of the presence of a bench may be explained by the participants in subgroup 4 being the most functionally limited, reporting higher levels of fear of falling, and being more likely to use a walking aid. These participants in particular may need a place to sit and rest while walking. This finding provides some support for the hypothesis raised by press-competence models that sensitivity to environmental factors is greater among more functionally limited persons [33]. Following this logic, one would expect older adults with functional limitations and fear of falling also to pay more attention to sidewalk evenness and the presence of obstacles, but this was not confirmed by our findings.

Overall, few differences in socio-demographic, functional and psychosocial characteristics, current level of walking and environmental perceptions between the subgroups were observed. Furthermore, the observed differences were small and suggest that environmental changes targeting improvements in sidewalk evenness will increase the appeal of streets for transportation walking among almost all Flemish older adults. One exception is that the presence of vegetation and benches may be particularly relevant for functionally limited older adults and service flat residents.

A key strength of the current study was the use of manipulated photographs which allowed us to examine the effects of manipulations in micro-scale environmental attributes on a street’s appeal for transportation walking under very controlled conditions. For example, distance to a destination has been shown to be strongly related to transportation walking. During the choice-task we controlled for this by instructing that distance to the friend’s home was similar across the two streets. Furthermore, the use of photographs enabled us to control the variations within an environmental attribute and the co-variations between environmental attributes. A further strength was the focus on identifying subgroups with different environmental preferences for transportation walking; this is important for modifying environments to the needs of different subgroups.

Besides these strengths, some limitations should be acknowledged. First, we examined the effects of environmental modifications on older adults’ preferences for transportation walking and not on actual transportation walking behavior. At this stage it is unclear whether modifying a street into a street with preferable environmental attributes (e.g. even sidewalks) will actually lead to more walking for transport. Therefore, our findings should be interpreted and used accordingly. Current study should not be considered an endpoint, but our findings provide valuable information for future studies that aim to examine effects of real environmental modifications on real transportation walking. Second, our sample was highly educated in comparison to the population of Flemish older adults; 36.3 % of participants had received tertiary education, while the population prevalence is 16.1 % [64]. This is not surprising since we recruited the majority of our sample online. However, there is currently no evidence that educational level (or other individual socio-economic characteristics) moderates the relationships between micro-scale environmental factors and transportation walking among older adults [21]. Furthermore, in the current study the four subgroups with different environmental preferences did not differ in educational level. Third, two of the identified subgroups included less than 90 participants while our sample size calculations had shown that a minimum of 90 participants were required. This may explain why fewer significant differences between importances and utilities were observed in subgroups three and four. Fourth, since traffic speed cannot be accurately depicted in a photograph, we used speed limit as a proxy for traffic speed. Speed limit may not correspond to actual traffic speed and the noise and exhausts generated by heavy traffic and fast-driving cars cannot be captured in a photograph. This might explain the limited importance observed for speed limit, traffic volume and traffic calming. Future research could use video material to better represent these attributes involving kinetic and auditory aspects.

## Conclusions

To conclude, our findings based on manipulated photographs can inform potential modifications of real-life settings regarding which environmental factors should be prioritized. Although we identified four subgroups with different environmental preferences, our results clearly show that the provision of even sidewalks should be considered a priority when developing such natural experiments aiming to stimulate older adults’ transportation walking. Our findings also indicate that special attention should be devoted to the presence of vegetation and benches in the surroundings of service flats. Natural experiments are needed to confirm whether the observed effects of manipulating photographed environmental attributes on a street’s appeal for transportation walking can be translated to real environments and actual transportation walking behavior.

## Additional file

Additional file 1:
**Assessment of environmental perceptions.** (PDF 772 kb)
