# Supplementary material for: Street characteristics preferred for transportation walking among older adults: a choice-based conjoint analysis with manipulated photographs
Source: Int J Behav Nutr Phys Act. 2016 Jan 16;13:6. doi: 10.1186/s12966-016-0331-8 (PMC4715277; doi:10.1186/s12966-016-0331-8)
Supplement: Additional file 1: — Assessment of environmental perceptions. (PDF 772 kb) [file 12966_2016_331_MOESM1_ESM.pdf]

<u>**Assessment of environmental perceptions**</u>

1. Solely considering the amount of vegetation in your street, which photograph best represents the situation in your street?

O No vegetation

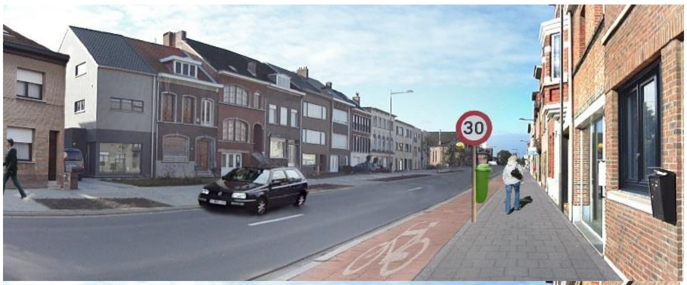

O Some vegetation

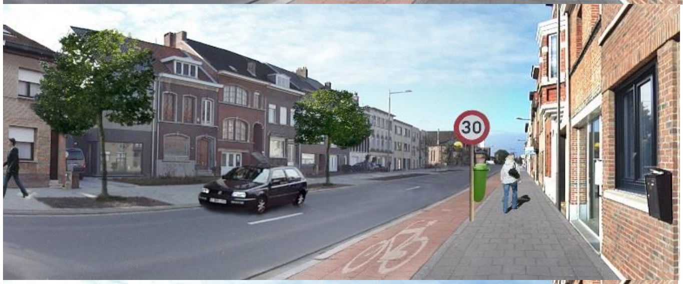

O A lot of vegetation

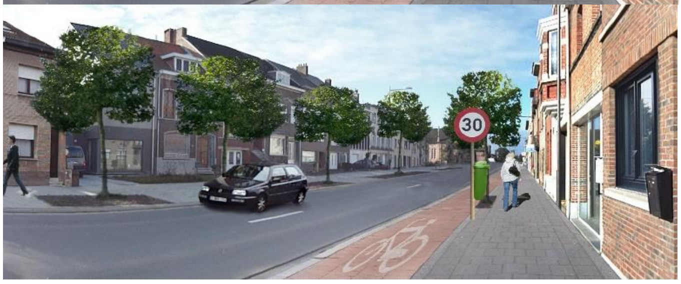

2. Solely considering the evenness of the sidewalk in your street, which photograph best represents the situation in your street?

O A very uneven sidewalk

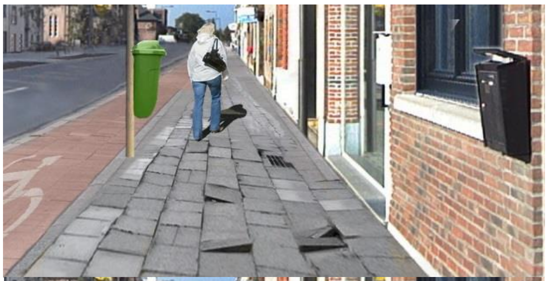

O A moderately uneven sidewalk

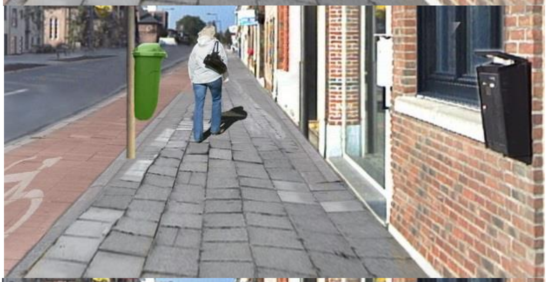

O An even sidewalk

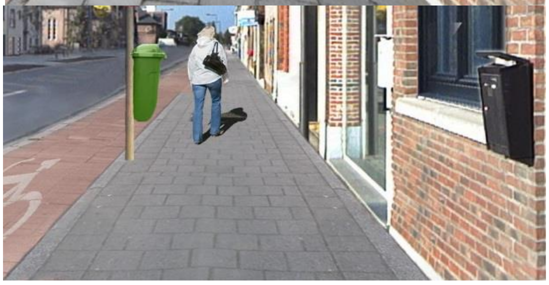

O There is no sidewalk in my street

3. Solely considering the presence of obstacle on the sidewalk in your street, which photograph best represents the situation in your street? (question was skipped if participants indicated previously that there was no sidewalk in their street)

O No obstacles on the sidewalk

O Obstacles present on the sidewalk (e.g. parked bicycles, traffic signage…)

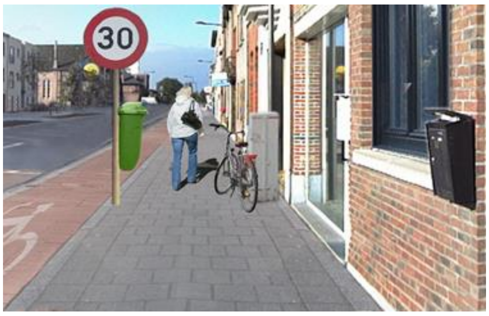

4. Solely considering the separation between the cycling path and sidewalk in your street, which photograph best represents the situation in your street? (question was skipped if participants indicated previously that there was no sidewalk in their street)

O There is no cycling path in my street, the sidewalk is only separated from motorized traffic by a curb

O There is no cycling path in my street, there is a separation between the sidewalk and motorized traffic (e.g., parked cars, a strip of grass, a shrub…)

O There is a cycling path in my street, there is no separation between the sidewalk and cycling path.

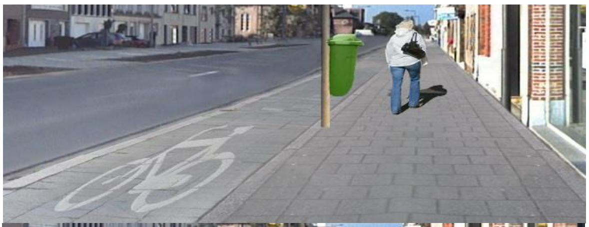

O There is a cycling path in my street, sidewalk and cycling path have a different color or there is a colored line between sidewalk and cycling path.

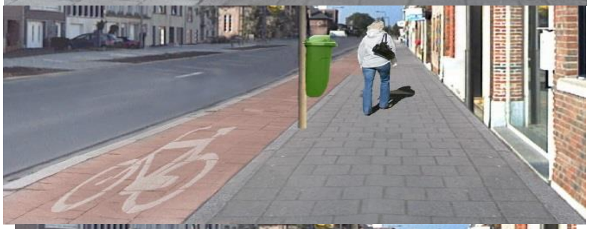

O There is a cycling path in my street and there is a real separation between sidewalk and cycling path (e.g. a curb, parked cars, a strip of grass, a shrub…)

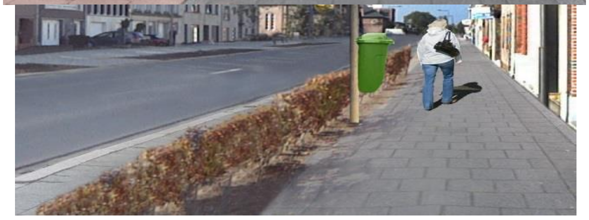

5. Solely considering the amount of traffic in your street, which photograph best represents the situation in your street?

O Light traffic

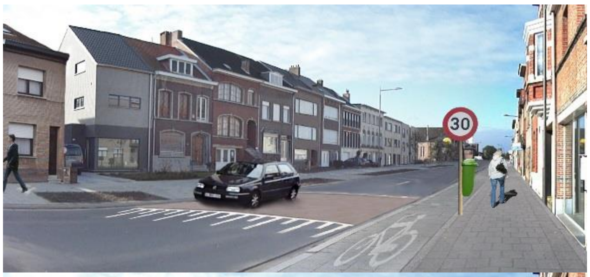

O Medium traffic

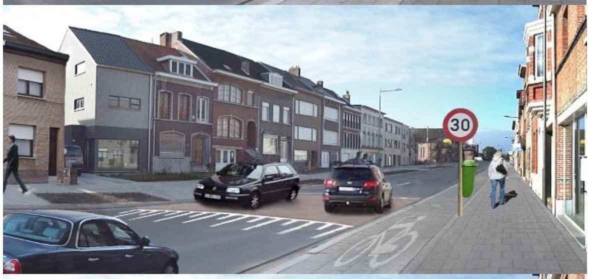

O Heavy traffic

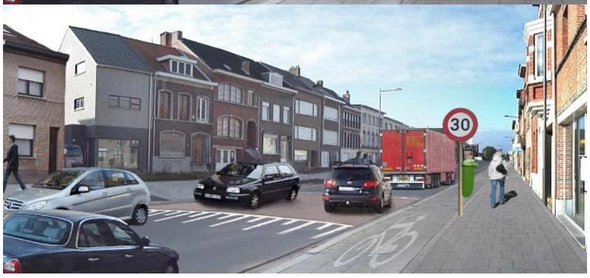

6. Solely considering the cleanliness of your street, which photograph best represents the situation in your street?

O Very clean

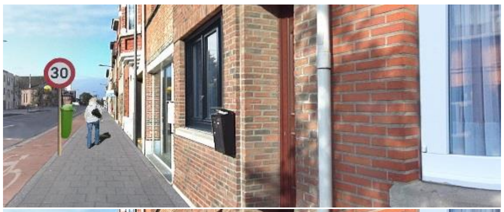

O Moderately clean

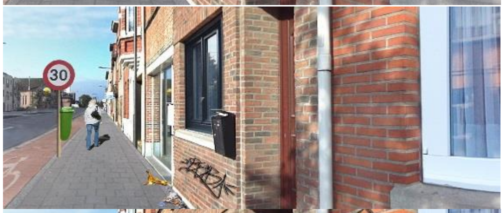

O Not clean at all

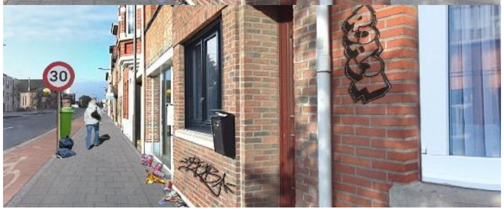

7. Solely considering the presence of benches in your street, which photograph best represents the situation in your street?

O No benches present

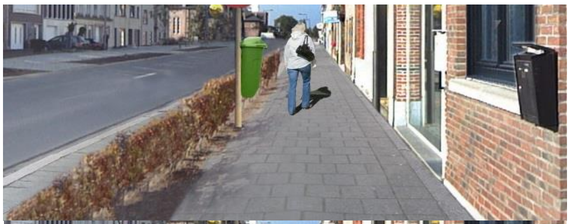

O Benches present

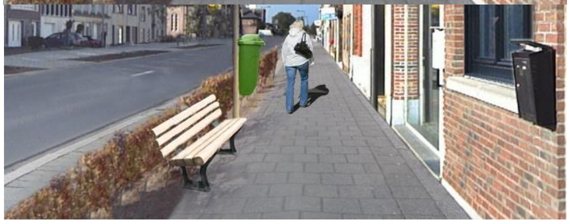

8. Solely considering the presence of traffic calming devices in your street (e.g. speed bumps, chicances), which photograph best represents the situation in your street?

O No traffic calming device present

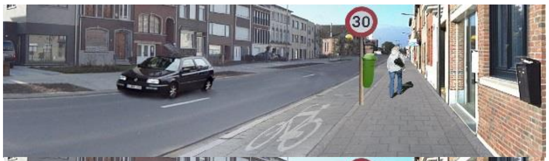

O Traffic calming device present

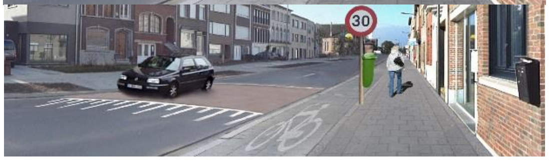

9. What is the speed limit in your street?

O 30 km/h

O 50 km/h

O 70 km/h

O 90 km/h
